# Identification of active gastric bleeding and achievement of endoscopic hemostasis via gel immersion endoscopy

**DOI:** 10.1055/a-2589-1469

**Published:** 2025-05-19

**Authors:** Yoshiki Morihisa, Yohei Yabuuchi, Yutaro Tokutomi, Tetsuro Inokuma

**Affiliations:** 1 26330 Department of Gastroenterology, Kobe City Medical Center General Hospital, Kobe, Japan

The presence of blood clots in the gastric body makes it difficult to identify the source of bleeding and achieve endoscopic hemostasis in patients with acute upper gastrointestinal bleeding 1 . Changing the patient’s position is useful for identifying the bleeding site, but it may be challenging depending on the patient's condition 2 .

This report describes a case in which the bleeding site was identified in the upper gastric body via gel immersion endoscopy, and endoscopic hemostasis was achieved by clipping 3 4 . Changing the patient’s position was difficult because of intubation and venoarterial extracorporeal membrane oxygenation (VA-ECMO).

A 78-year-old woman underwent artificial replacement of a thoracoabdominal aortic aneurysm and returned to the intensive care unit under endotracheal intubation. Two days after surgery, the patient developed postoperative septic shock, refractory atrial fibrillation, and right heart failure. Therefore, intra-aortic balloon pumping implantation and VA-ECMO were performed. Six days after the initiation of ECMO, the patient was referred to our department for hematemesis.

Contrast-enhanced computed tomography (CECT) showed extravasation of contrast material in the posterior wall of the upper gastric body ( Fig. 1 ). The gastric body was filled with a large amount of clotted blood and endoscopic suction was ineffective. Identifying the bleeding site was difficult due to blood clots in the gastric body ( Fig. 2 ). The injection of gel (Viscoclear; Otsuka Pharmaceutical Factory) created a space between the gastric wall and the blood clots. This technique provides a clear field of view to avoid clotting ( Fig. 3 , Video 1 ). Active bleeding was observed on the posterior wall of the upper gastric body, consistent with CECT findings ( Fig. 4 ). Endoscopic hemostasis was achieved by clipping ( Fig. 5 ). Gel immersion endoscopy is useful when the source of bleeding cannot be identified due to the presence of large blood clots in the gastric body and difficulties in changing the patient’s position.

**Fig. 1:**
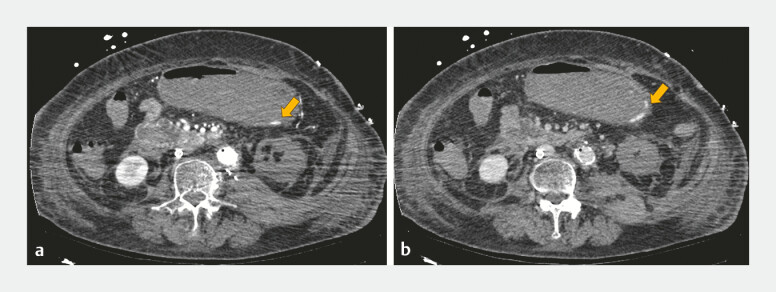
Contrast-enhanced computed tomography showed extravasation of contrast material in the posterior wall of the upper gastric body. **a** Arterial phase. **b** Venous phase.

**Fig. 2:**
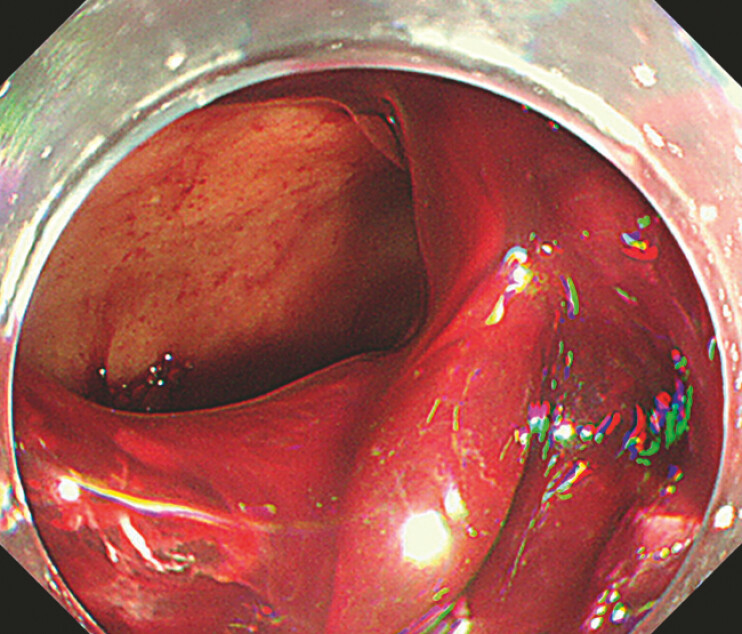
The presence of blood clots in the gastric body made it difficult to identify the source of bleeding.

**Fig. 3:**
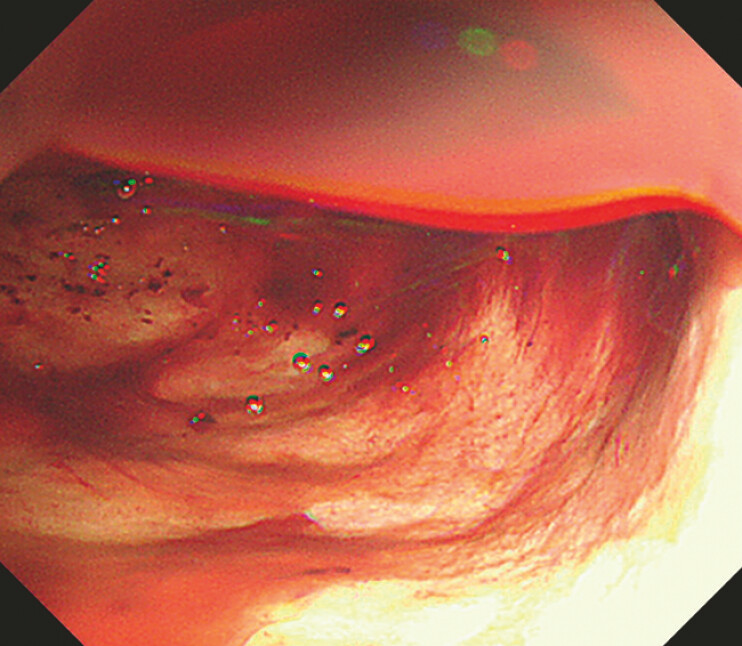
The injection of gel created a space between the gastric wall and the blood clots, providing a clear field of view to avoid clotting.

**Fig. 4:**
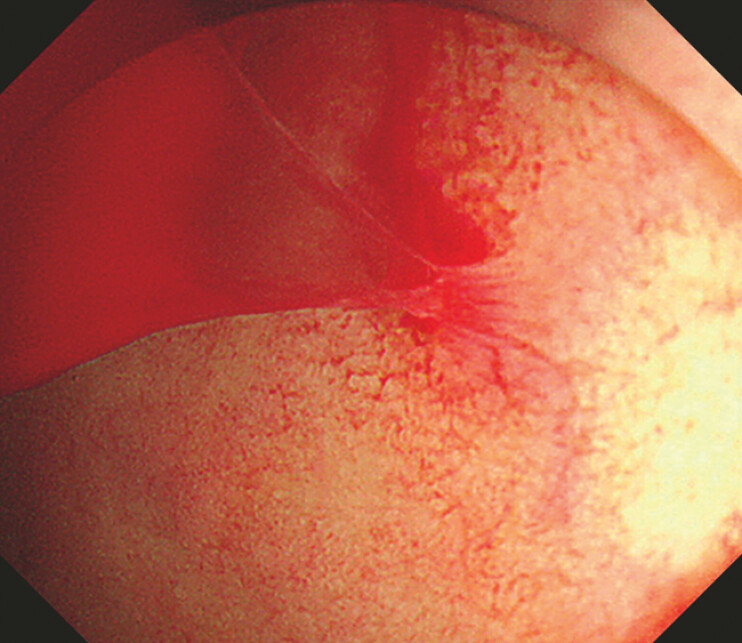
Active bleeding was observed on the posterior wall of the upper gastric body.

**Fig. 5:**
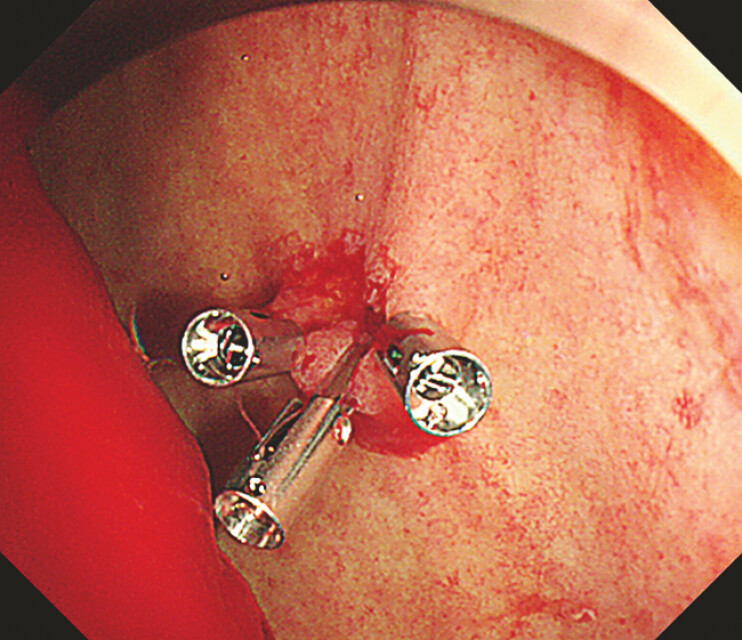
Endoscopic hemostasis was achieved by clipping.

Identification of active gastric bleeding and achievement of endoscopic hemostasis by gel immersion endoscopy in a situation in which changing the patient’s position was difficult.Video 1

Endoscopy_UCTN_Code_TTT_1AO_2AD

## Notes

Endoscopy E-Videos https://eref.thieme.de/e-videos
*E-Videos* is an open access online section of the journal *Endoscopy* , reporting on interesting cases and new techniques in gastroenterological endoscopy. All papers include a high-quality video and are published with a Creative Commons CC-BY license. Endoscopy E-Videos qualify for HINARI discounts and waivers and eligibility is automatically checked during the submission process. We grant 100% waivers to articles whose corresponding authors are based in Group A countries and 50% waivers to those who are based in Group B countries as classified by Research4Life (see: https://www.research4life.org/access/eligibility/ ). This section has its own submission website at https://mc.manuscriptcentral.com/e-videos .
